# Abiotic Stress Tolerance of Charophyte Green Algae: New Challenges for Omics Techniques

**DOI:** 10.3389/fpls.2016.00678

**Published:** 2016-05-20

**Authors:** Andreas Holzinger, Martina Pichrtová

**Affiliations:** ^1^Unit of Functional Plant Biology, Institute of Botany, University of Innsbruck, InnsbruckAustria; ^2^Department of Botany, Faculty of Science, Charles University in Prague, PragueCzech Republic

**Keywords:** transcriptomics, proteomics, metabolomics, UV irradiation, desiccation, phylogenomic analysis

## Abstract

Charophyte green algae are a paraphyletic group of freshwater and terrestrial green algae, comprising the classes of Chlorokybophyceae, Coleochaetophyceae, Klebsormidiophyceae, Zygnematophyceae, Mesostigmatophyceae, and Charo- phyceae. Zygnematophyceae (Conjugating green algae) are considered to be closest algal relatives to land plants (Embryophyta). Therefore, they are ideal model organisms for studying stress tolerance mechanisms connected with transition to land, one of the most important events in plant evolution and the Earth’s history. In Zygnematophyceae, but also in Coleochaetophyceae, Chlorokybophyceae, and Klebsormidiophyceae terrestrial members are found which are frequently exposed to naturally occurring abiotic stress scenarios like desiccation, freezing and high photosynthetic active (PAR) as well as ultraviolet (UV) irradiation. Here, we summarize current knowledge about various stress tolerance mechanisms including insight provided by pioneer transcriptomic and proteomic studies. While formation of dormant spores is a typical strategy of freshwater classes, true terrestrial groups are stress tolerant in vegetative state. Aggregation of cells, flexible cell walls, mucilage production and accumulation of osmotically active compounds are the most common desiccation tolerance strategies. In addition, high photophysiological plasticity and accumulation of UV-screening compounds are important protective mechanisms in conditions with high irradiation. Now a shift from classical chemical analysis to next-generation genome sequencing, gene reconstruction and annotation, genome-scale molecular analysis using omics technologies followed by computer-assisted analysis will give new insights in a systems biology approach. For example, changes in transcriptome and role of phytohormone signaling in *Klebsormidium* during desiccation were recently described. Application of these modern approaches will deeply enhance our understanding of stress reactions in an unbiased non-targeted view in an evolutionary context.

## Charophyte Algae in Terrestrial Environments

Charophyte green algae are a diverse paraphyletic assemblage of strictly freshwater algae (Leliaert et al., 2012) comprising about 100 genera. We can distinguish ‘advanced charophytes’ (Zygnematophyceae, Coleochaetophyceae, Charophyceae) recently designated as ZCC clade (de Vries et al., 2016) and ‘basal charophytes’ (Klebsormidiophyceae, Chlorokybophyceae, Mesostigmatophyceae), designated as KCM clade (de Vries et al., 2016). However, charophyte green algae are not only restricted to aquatic habitats. Terrestrial forms occur in the classes Chlorokybophyceae (Lewis and McCourt, 2004), the Klebsormidiophyceae (e.g., Karsten et al., 2010; Karsten and Holzinger, 2012), the Zygnematophyceae (e.g., Lewis and McCourt, 2004; Holzinger et al., 2009, 2010) and the Coleochaetophyceae (Graham et al., 2012, 2013) and viable airborne cells of various charophyte algae were also reported (Sharma et al., 2007).

Colonization of moderately moist habitats in the proximity of water by the charophyte algal ancestor of land plants and its gradual transition to drylands has been suggested by Becker and Marin (2009). Recently, several reports have demonstrated the close relationship of land plants and Zygnematophyceae that are currently viewed as sister lineages (Becker and Marin, 2009; Wodniok et al., 2011; Timme et al., 2012; Becker, 2013; Zhong et al., 2014, 2015; Delaux et al., 2015). At present, Zygnematophyceae dominate in various stressful habitats. For example, desmids are typically found in acidic bogs (Štástný, 2010), *Zygogonium ericetorum* is a common member of temperate biological soil crust (Hoppert et al., 2004) and *Mesotaenium berggrenii* and *Ancylonema nordenskiöldii* live on the surface of glaciers on bare ice (Remias et al., 2009, 2012a,b).

**FIGURE 1:**
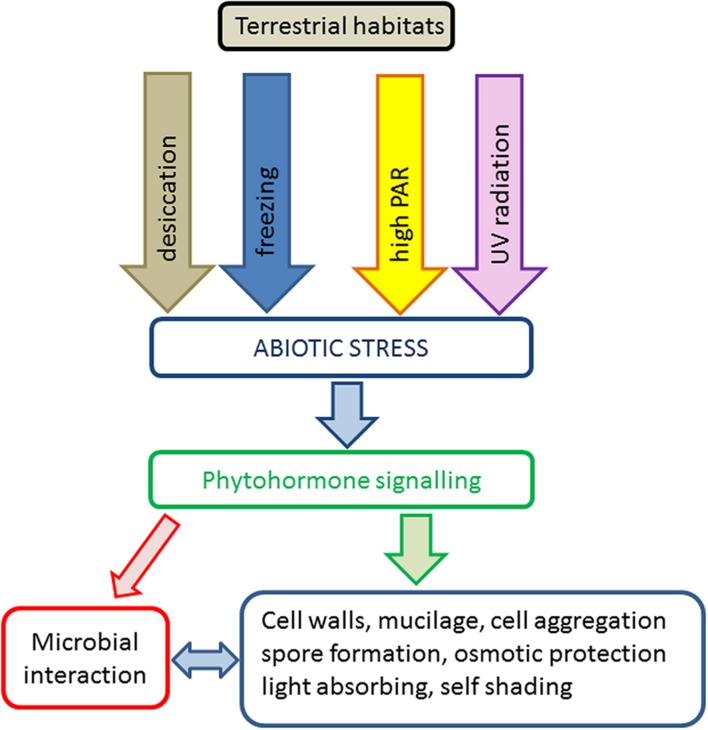
**Schematic representation of abiotic stress factors and tolerance strategies in charophyte green algae in terrestrial habitats**.

Transition to terrestrial habitats is connected with frequent exposure to naturally occurring abiotic stress scenarios like desiccation, freezing and high PAR and UV radiation. The effects of these stresses on ultrastructure, photosynthesis and ecology in green algae have recently been reviewed (Holzinger and Lütz, 2006; Holzinger and Karsten, 2013; Karsten and Holzinger, 2014). In the present review, we mainly focus on the current knowledge about abiotic stress tolerance mechanisms known in terrestrial members of the charophyte algae as model systems to study terrestrialization events. We include important classical studies based on traditional methods as well as new understanding derived from recent transcriptomic and genomic datasets. In general, stress tolerance strategies are summarized in a simplified schema (**Figure 1**).

## Self-Protection

The morphologically most complex group of charophyte algae, the class Charophyceae, is restricted solely to aquatic environment (Leliaert et al., 2012). On the other hand, terrestrial charophytes are characterized by a very simple thallus, unicellular or filamentous which, however, usually aggregate into colonies, multi-layered mats or biofilms. This growth pattern belongs to common stress avoidance strategies and provides protection from multiple stresses at the same time. While the outer layers are fully exposed to the environment and susceptible to damage, at the same time they efficiently protect the cells underneath by their water-holding and screening capacity. In addition, aeroterrestrial algae are also components of biological soil crusts, microecosystems containing also bacteria, Cyanobacteria, fungi, lichens, mosses, and anorganic particles where the whole community can profit from protection provided by an individual member (Belnap and Lange, 2001).

*Chlorokybus atmophyticus*, the only known member of the class Chlorokybophyceae, occurs in subaerial habitats and is characterized by sarcinoid colonies with groups of cells embedded in soft mucilage (Škaloud, 2009).

Freshwater *Coleochaete* usually forms flat epiphytic disk or cushion-like thalli, composed by densely branched filaments. However, when grown in aero-terrestrial conditions, namely on agar or sand, it markedly changes its morphology and growth habitus. It forms multistratose clusters of thick walled cells with acetolysis resistant autofluorescent cell wall components (Graham et al., 2012).

Aeroterrestrial members of the class Klebsormidiophyceae form multi-layered biofilms on soil or other aeroterrestrial substrata (Karsten and Rindi, 2010; Karsten et al., 2013). This provides above all self-shading and photoprotection of individual filaments inside the mat which can be even enhanced by soil particles interwoven within the mats (Karsten and Holzinger, 2012; Karsten et al., 2013). The importance of self-shading is reflected by generally low light requirements for photosynthesis in *Klebsormidium* that were repeatedly shown (Karsten and Rindi, 2010; Karsten et al., 2013).

Mat-forming growth is also typical for filamentous Zygnematophyceae. Filaments usually start to grow at the bottom of a pool and when enough biomass is produced, oxygen bubbles trapped within it carry the mat to the surface of the pool (Graham et al., 1995). The top layers are then fully exposed to solar radiation which leads to their bleaching. Berry and Lembi (2000) measured irradiance below the *Spirogyra* mat to be more than 30 times lower than at the mat surface. Moreover, the photosynthetic rate measured under experimental conditions was higher at lower irradiance showing that underlying filaments were exposed to more optimal irradiances (Berry and Lembi, 2000). Similarly, the low light adaptation in *Zygnema* is also usually explained by photoprotection provided by multi-layered mats (Holzinger et al., 2009; Herburger et al., 2015).

*Spirogyra* mats were even shown to be able of phototactic movement at low light conditions. The filaments align toward the light source and when they touch other filaments, they glide along each other, form bundles and move toward the light source by repeated rolling and stretching (Kim et al., 2005).

**FIGURE 2:**
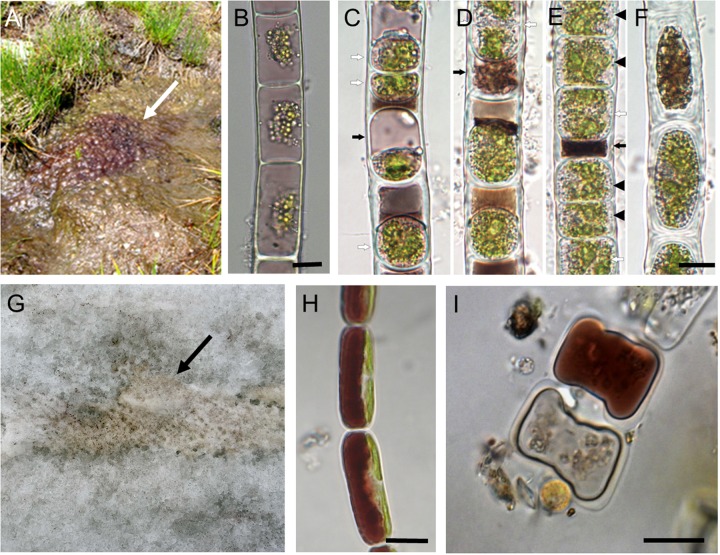
**Zygnematophyceae with purple vacuolar pigmentation. (A–F)** *Zygogonium ericetorum*; **(G–I)** *Ancylonema nordenskiöldii*. **(A)** Habitat showing purple coloration (arrow), **(B)** vegetative filament, **(C–E)** aplanospore formation, white arrows indicate enlarged aplanospores with cell wall cleavage, black arrows point to compacted brownish residues, black arrowheads illustrate germinated aplanospores, **(F)** thick walled akinetes, **(G)** habitat on the surface of a glacier, arrow indicates coloration by algae **(H)** vegetative filament with purple coloration of the vacuole, **(I)** conjugation stage. Bars **(B–F,H,I)** 10 μm. **(A,B)** Reprinted from Aigner et al. (2013), **(C–F)** reprinted from Stancheva et al. (2014), **(G–I)** reprinted from Remias et al. (2012a) with permission of Springer Business Media.

The differentiation of mat layers is best developed in terrestrial filamentous conjugating green alga *Z. ericetorum*. *Zygogonium ericetorum* produces two types of cells termed green and purple morphs (**Figures 2A,B**), (Aigner et al., 2013). The purple morph of the top layers is better protected from high irradiation and helps to shade the green morph underneath which is in turn less sensitive to desiccation (Holzinger et al., 2010; Aigner et al., 2013).

## Formation of Specialized Cells

Formation of specialized stress-tolerant cells as a part of the life cycle is a widespread strategy for survival of unfavorable conditions and is widely known from many different groups of algae and other protists.

The first type of stress tolerant cells are dormant zygotes, i.e., resting cells that are developed as a result of sexual reproduction. Within Charophyta, such cells have been described in Coleochaetophyceae, Charophyceae, and Zygnematophyceae. Dormant zygotes usually require a period of dormancy before germination. Dormancy can be broken by a change in environmental conditions (temperature, light) or by addition of gibberellic acid (Sederias and Colman, 2007; Agrawal, 2009). Possible roles in phytohormone stress sensing and signal transduction are discussed below.

In Charophyceae, the dormant zygotes are termed oospores. Oospores are the overwintering stages that can survive anoxic conditions on lake bottoms. They are the only desiccation tolerant stages in the life cycle of Charophyceae and thus guarantee their survival in habitats that dry out for several years (Proctor, 1967; Leliaert et al., 2012). The young zygote secretes a thick cell wall and the inner walls of cortical cells thicken and become encrusted with lime. Calcified envelopes of the zygotes are known as “gyrogonites” (Leliaert et al., 2012).

Dormant zygotes, termed zygospores, that contain acetolysis resistant material are typical feature of the life cycle of Coleochaetophyceae (Delwiche et al., 1989) and Zygnematophyceae (e.g., *Zygnema* sp.: Stancheva et al., 2012, *Spirogyra* sp.: Stancheva et al., 2013 and *Z. ericetorum*: Stancheva et al., 2016). Cell walls of Zygnematophyceae zygospores consist of three major layers. Endospore is the colorless inner layer. The middle layer, termed mesospore, is crucial for stress tolerance as it contains enzyme- and acetolysis-resistant sporopollenin-like material. Exospore is at least partially enzyme-soluble, colorless and sometimes also sculptured (Ashraf and Godward, 1980). Zygospores can be spherical, ellipsoid, rectangular, or lenticular and may be colored yellow, brown, purple, blue, or black (Kadlubowska, 1984). Most of the morphological characters important for traditional species determination are on the mesospore, where a blue vs. brown mesospores were suggested to separate different phylogenetic clades (Stancheva et al., 2012).

The other type of specialized cells is not formed during the sexual process, even though morphologically they can be very similar to the sexual spores. In Zygnematophyceae, mostly in filamentous Zygnematales, several such cell types were described. Parthenospores result from incomplete conjugation; they form directly from gametes that failed to find a compatible sexual partner (Kadlubowska, 1984). They are smaller and more rounded than zygospores (Poulíčková et al., 2007). Aplanospores are formed inside vegetative cells when protoplasts shrink and are enclosed by a complete new cell wall, independent of the original wall (Kadlubowska, 1984). Their formation was recently described in *Z. ericetorum* (**Figures 2C–F**), (Stancheva et al., 2014). Finally, akinetes develop directly from vegetative cells by thickening of the cell wall by deposition of wall material at the inner surface of the vegetative cell wall (Kadlubowska, 1984). In some species of *Zygnema*, the cell walls of mature akinetes can have a similar structure, ornamentation, and coloration to that of the zygospores (Kadlubowska, 1984). For morphology and light microscopy images of zygospores and akinetes, see Stancheva et al. (2012).

In contrast, many algae from stressful environments do not form any specialized stages and survive environmental stresses in vegetative state (Agrawal, 2009). Such cells are not dormant, so they can resume their physiological activity and growth immediately under restoring of favorable conditions (Agrawal, 2009). Nevertheless, vegetative cells in natural or experimentally induced stressful conditions differ from growing, non-stressed vegetative cells in many ways. They are usually characterized by storage product accumulation, thick cell wall, reduced physiological activity and ceased cellular division. Traditionally, such cells were sometimes termed ‘akinetes’ or even ‘winter forms’ in *Micrasterias* (Meindl et al., 1989). To avoid the confusion with ‘akinetes’ in *Zygnema* which are true specialized cells with zygospore-like cell walls, the term ‘pre-akinete’ or ‘mature cell’ has also been introduced by some authors for these hardened, stress tolerant vegetative cells (Fuller, 2013; Pichrtová et al., 2014a,b, 2016a; Herburger et al., 2015). The term ‘mature cell’ refers to the typical occurrence of such cells in old, starved cultures.

**FIGURE 3:**
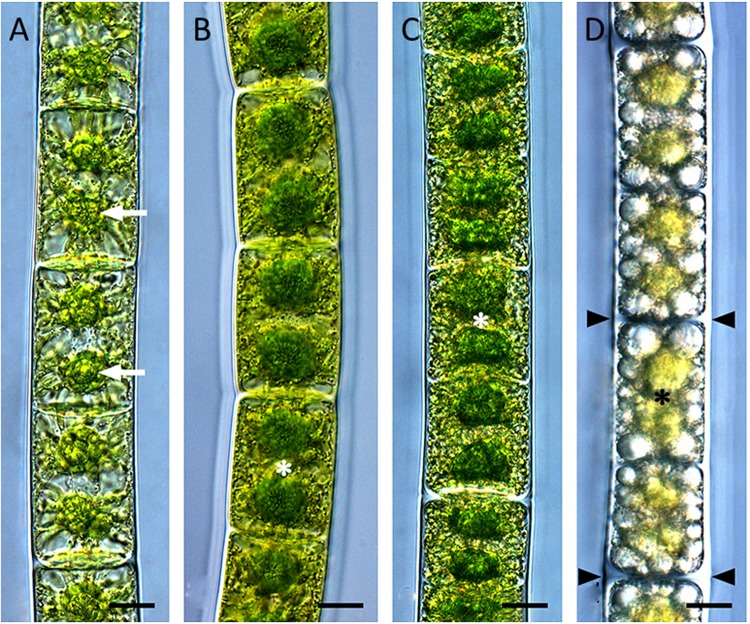
**Pre-akinete formation in *Zygnema* sp. Saalach. (A)** 1 month, **(B)** 6 months, **(C)** 9 months, **(D)** 15 months old cultures. Arrows: pyrenoids, asterisks: nuclei, arrowheads: thickened cell walls. Bars 10 μm. Reprinted from Herburger et al. (2015).

Mature cells (pre-akinetes) are key elements for survival of *Zygnema* in stressful environment where sexual reproduction and zygospore formation is very rare. Pre-akinetes of Arctic and Antarctic *Zygnema* survived when exposed to osmotic stress (Pichrtová et al., 2014a), desiccation (Pichrtová et al., 2014b), UV radiation (Holzinger et al., 2009), freezing (Hawes, 1990), and they were even observed to be the overwintering stages (Pichrtová et al., 2016b). Formation of mature cells (pre-akinetes) during aging of the cultures was tracked in cultures of Alpine strains of *Zygnema* (**Figure 3**), (Herburger et al., 2015); old cultures survived desiccation at 84% RH and had in general lower rETR max (Herburger et al., 2015). However, only after previous acclimation by slow desiccation (induced either by controlled desiccation at high relative humidity or by pre-cultivation on agar), the pre-akinetes were able to survive rapid desiccation in air at a relative humidity of 10% (Pichrtová et al., 2014b).

Glacier surface belongs to the most extreme habitats on Earth and it is interesting that the only eukaryotic algae that live there belong to the class Zygnematophyceae. Field populations of these algae, namely species *A. nordenskioeldii* (**Figures 2G–I**), (Remias et al., 2012a) and *M. berggrenii*, are also composed of vegetative cells; in the field no spores or cysts are produced even for overwintering (Remias et al., 2009, 2012a), however, conjugation has been observed in *A. nordenskioeldii* in field collected samples that were transferred to the laboratory (**Figure 2I**).

Members of the class Klebsormidiophyceae are also stress tolerant in their vegetative cells. Morison and Sheath (1985) showed a change in cellular morphology in desiccation tolerant *K. rivulare* resembling pre-akinete formation in *Zygnema*. Upon desiccation, carbohydrate and lipid contents increased and protein content decreased (Morison and Sheath, 1985). Many papers have recently been published that investigated various strains of *Klebsormidium*, *Interfilum*, *Hormidiella*, and *Entransia* in relation to desiccation stress (Karsten et al., 2010, 2013, 2014; Karsten and Holzinger, 2012; Herburger et al., 2016a). All species investigated were able to survive experimental desiccation and even the aquatic *Entransia* coped with low water availability better than other freshwater algae (Herburger et al., 2016a). Alpine strains of *Klebsormidium* were also markedly resistant to osmotic stress (Kaplan et al., 2012). Moreover, most of the investigated *Klebsormidium* sp. strains also survived freezing at -40°C (Elster et al., 2008).

## Cell Wall Structure and Composition in Relation to Stress Tolerance

Cell wall composition of charophyte green algae has recently been investigated extensively, particularly before the background that they are thought to be closest living relatives to land plants (Mikkelsen et al., 2014). Charophyte green algae have cell walls containing polymers with remarkable similarity to cellulose, pectins, hemicelluloses, arabinogalactan proteins (AGPs), extensin, and lignin present in embryophyte walls (Sørensen et al., 2011; Domozych et al., 2012). Charophyte green algae contain orthologous genes in the cellulose synthetase (*cesA*) family, as well as in the *cesA*-like families *cslC* and *cslD* as analyzed by a survey of genomes and transcriptomes (Yin et al., 2014). Recent review articles summarize cell wall composition of charophytes (Popper and Tuohy, 2010; Popper et al., 2011, 2014; Domozych et al., 2012; Sørensen et al., 2012; Domozych and Domozych, 2014). We will focus on the cell wall components that are involved in desiccation and irradiation stress tolerance. The cell walls of streptophytic green algae have been suggested to be a defining structure that enabled green algal ancestors to colonize land (Mikkelsen et al., 2014). However, what are the traits that enabled this colonization? All charophyte green algae are poikilohydric organisms, they equilibrate with the surrounding relative air humidity, and do not have mechanisms that protect from desiccation. However, some cell wall components might have the sole function of prolonging the water holding capacities for algae that were exposed to desiccating conditions.

One of the major differences in early-divergent chlorophyte and prasinophyte algae genomes is the occurrence of a low number of glycosyl transferases (GTs), whereas land plants contain hundreds of GTs. Now there is genetic evidence that many of the core cell wall polysaccharides have their origin in charophyte green algae (Domozych et al., 2012). These include mannan, xyloglucan, xylan, pectin, and arabino-galactan proteins (Mikkelsen et al., 2014). Most important for stress tolerance are pectic proteins, which have been extensively studied in the mesotaeniaceae *Netrium* and the desmidiacean green algae *Closterium*, *Penium*, and *Micrasterias* (e.g., Domozych and Rogers-Domozych, 1993; Brosch-Salomon et al., 1998; Domozych et al., 2007; Eder et al., 2008; Eder and Lütz-Meindl, 2010). While the chemical structure of the detected homogalacturonans (α-D-1,4-galacturonic acid) might vary in the degree of methyl esterification, relevant for the function of these compounds during developmental processes. When incorporated into mucilage, particularly the water holding capacities of these layers are ecologically relevant in habitats with fluctuating water regimes. Formation of mucilage layers is widespread in Zygnematophyceae. For example, in *Zygnema irregulare* formation of secondary pectic layers was observed during prolonged cultivation on solid medium (Fuller, 2013). In the desmid *Micrasterias denticulata* mucilage layers have been described and characterized (e.g., Oertel et al., 2004; Wanner et al., 2013). Additionally, pectic layers have another great benefit in charophyte green algae – allowing to form multicellular organisms, a clear prerequisite of success for plants on land (Domozych and Domozych, 2014). Moreover, a mucilage layer has also been suggested to play a significant role in UV protection (Lütz et al., 1997), however, UV screening compounds have not directly been identified in the mucilage. Recently, homogalacturonans have been described as ancient streptophyte feature, albeit secondarily lost in *Klebsormidium* (O’Rourke et al., 2015). Members of the Klebsormidiales have indeed many differences concerning their cell walls (Mikhailyuk et al., 2014), when compared to later branching charophytes.

**FIGURE 4:**
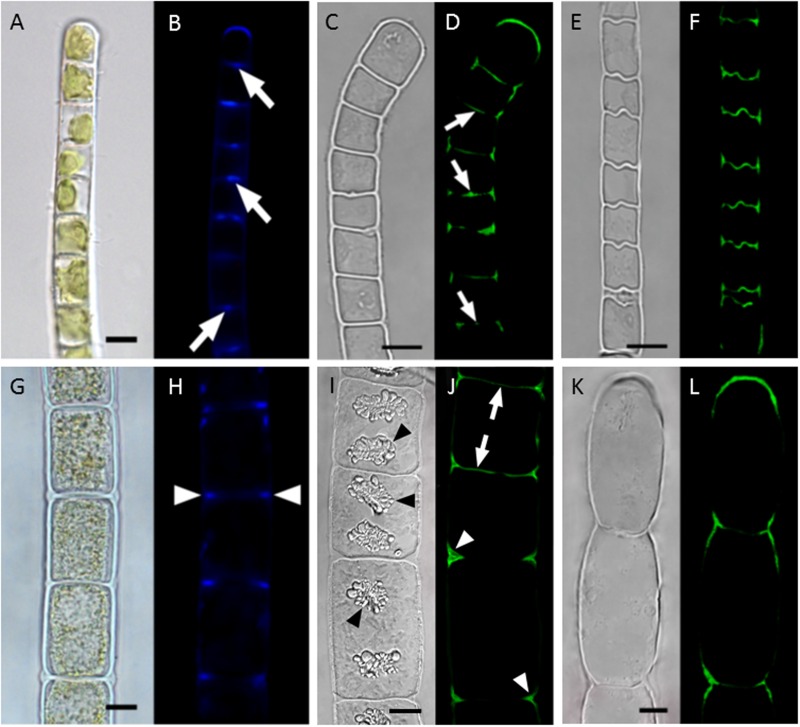
**Localization of callose in cell walls of *Klebsormidium crenulatum***(A–F)** and *Zygnema* sp. Saalach (G,H). (A,B)** Aniline blue staining, **(C,D)** staining of turgescent cell with antibody 400-2, **(E,F)** staining of desiccated cells with antibody 400-2, **(G,H)** aniline blue staining, **(I,J)** staining of turgescent cells with antibody 400-2, **(K,L)** staining of desiccated cell with antibody 400-2. Arrows: cross walls, arrowheads: cell corners. Bars 10 μm. reprinted from Herburger and Holzinger (2015).

The localization and function of callose, a β-D-1,3-glucan has been investigated in *Klebsormidium* and *Zygnema* (Herburger and Holzinger, 2015). Callose as a cell wall flexibilizing compound can protect cell walls from desiccation induced damage, when they follow the shrinkage of the protoplasts. It was previously shown by biochemical methods, that a high abundance of this compound was found in the early branching charophyte *Klebsormidium* (Sørensen et al., 2011). Upon localization this flexibilizing compound is predominately found in mechanically strained areas of the cell wall (Herburger and Holzinger, 2015). In *Klebsormidium* the absolute content of callose is significantly increased upon desiccation stress applied for up to 3 h. Localization of callose in *Klebsormidium* demonstrated that particularly the cross cell walls and the cell walls of the terminal cells contain high amounts of this compound, and thus can fold and follow the desiccation induced volume reduction (**Figures 4A–F**), (Herburger and Holzinger, 2015). In contrast, the later branching *Zygnema*, did not change the callose content upon desiccation stress. However, callose can still be localized in the cell walls of *Zygnema*, particularly in the cell corners, that are exposed to the most severe strain upon desiccation stress (**Figures 4G–L**). Desiccation induced volume changes have recently been investigated in *Klebsormidium* and *Zygnema* at different relative humidities, where *Zygnema* showed a more drastic volume reduction at the lowest RH tested (Lajos et al., 2016). Interestingly the more rigid, cellulose-rich secondary walls of *Zygnema* tolerate this stress without being damaged.

The occurrence of fossilizable biomacromolecules resistant to chemical processes in spores of charophyte green algae has been reported repeatedly (Versteegh and Blokker, 2004). The green autofluorescence of Zygnematophyceae zygospores is characteristic for sporopollenin-like material (algaenan) that differs from true sporopollenin of pollen grains in chemical composition and in biochemical pathway leading to its production (Versteegh and Blokker, 2004; Poulíčková et al., 2007). While sporopollenin is synthesized in phenylpropanoid pathway, the acetate–malate pathway leads to algaenans (Versteegh and Blokker, 2004). Similarly, in *Coleochaete* the inner zygote cell wall layers have been described to contain material similar in ultrastructural appearance and chemistry to sporopollenin (Delwiche et al., 1989; Kroken et al., 1996). Sporopollenin is polymerized from hydroxylated fatty acids and phenolics, its occurrence predated lignin (summarized in Weng and Chapple, 2010). Sporopollenin and algaenans in the cell walls provides protection from both desiccation and UV radiation. The occurrence of ‘lignin-like’ phenolic compounds were described in walls of vegetative cells of *Coleochaete* (Delwiche et al., 1989). However, many of these early findings were based on crude detection methods and have to be discussed critically (Weng and Chapple, 2010).

## Physiological Protection of the Photosynthetic Apparatus

One of the key targets of radiation (high PAR and UV) as well as desiccation and temperature stress is photosynthesis. The effects of desiccation on photosynthesis of green algae were recently summarized (Holzinger and Karsten, 2013; Karsten and Holzinger, 2014). Photosynthetic strategies of desiccation tolerant organisms were recently summarized by Fernandez-Marin et al. (2016). It has been shown, that dehydration directly affects the electron transport chains in the thylakoid membranes. The loss of water in the presence of light increases the risk of chlorophyll overexcitation, which can in turn generate oxygen radicals and reactive oxygen species (ROS). These compounds have various negative effects that will not be further discussed in this review. To compensate these effects, tolerant organisms up-regulate photoprotection mechanisms (Fernandez-Marin et al., 2016). The regulation of the photosynthetic electron transport rates and protection through photoinhibition is crucial for survival (e.g., Roach and Krieger-Liszkay, 2014). UV-B radiation has been shown to induce similar photo-oxidative stress in charophyte green algae (Germ et al., 2009; Karsten and Holzinger, 2014). The shift form LHCSR (light-harvesting complex stress-related) in chlorophytes to PSBS (photosystem II subunit S)-dependent non-photochemical quenching (NPQ) in charophytes has been demonstrated in ZCC-clade genera (*Chara*, *Zygenma*, and *Cosmarium;* Gerotto and Morosinotto, 2013). While *Zygnema* and *Cosmarium* showed a fast NPQ induction upon light exposure, *Klebsormidium* and *Interfilum* as representatives of the KCM-clade had a much slower induction. The only exception was *Mesotaenium*, where little NPQ induction was found (Gerotto and Morosinotto, 2013), however, in this genus different strategies like abundant protection by phenolic compounds are established (see below).

Numerous publications have investigated photosynthetic performance under laboratory controlled conditions in Klebsormidiophyceae (Karsten et al., 2010, 2013, 2014, 2015; Karsten and Holzinger, 2012) and in Zygnematophyceae (Pichrtová et al., 2013, 2014b; Herburger et al., 2015). Some information on photosynthesis parameters and light acclimation are available in Characeae (Küster et al., 2000; Vieira and Necchi, 2003). In contrast, little is known on other charophyte green algae according their photosynthetic performance.

**FIGURE 5:**
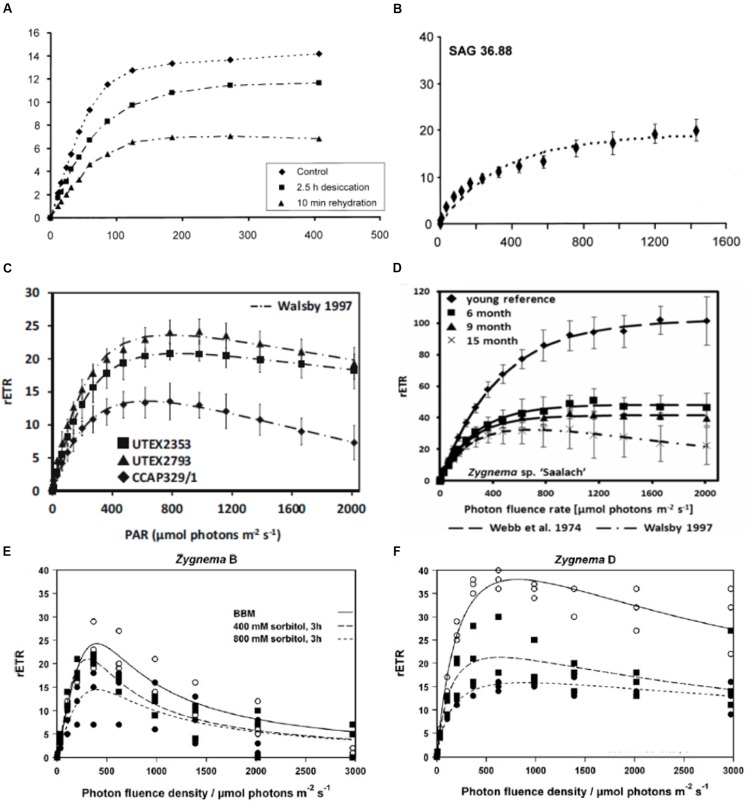
**Relative electron transport rates in different charophyte green algae. (A)** *Klebsormidium crenulatum*, (SAG 2415, desiccation and rehydration, reprinted from Karsten et al., 2010 with permission of Wiley and Sons.), **(B)** *Interfilum* (SAG 36.88), reprinted from Karsten et al. (2014), **(C)** *Entransia fimbriata* (UTEX2353, UTEX2793) and *Hormidiella attenuata* (CCAP329/1) reprinted from Herburger et al. (2016a), **(D)** *Zygnema* sp. Saalach (SAG 2419, various culture ages, reprinted from Herburger et al., 2015), **(E)** arctic *Zygnema* sp. ‘B’ (CCALA976, **(F)** Antarctic *Zygnema* sp. ‘D’ (**E,F**) reprinted from Kaplan et al. (2013).

Many physiological data are available for the genus *Klebsormidium* (Karsten et al., 2010, 2013, 2015; Karsten and Holzinger, 2012). Regardless of strain and clade, *Klebsormidium* shows a broad light amplitude, the relative electron transport rate did not show photoinhibition up to 500 μmol photons m^2^ s^-1^ in *Klebsormidium crenulatum* (**Figure 5A**), (Karsten et al., 2010). Very low light compensation points in the range 1.8–5.7 μmol photons m^2^ s^-1^ were found for different *Klebsormidium* species (Karsten et al., 2010; Karsten and Holzinger, 2012). The maximum photosynthetic oxygen production ranged from 37.9 μmol O_2_ h^-1^ mg^-1^ chl a in *K. crenulatum* (Karsten et al., 2010), 75.9 μmol O_2_ h^-1^ mg^-1^ chl a in *K. flaccidum* (Karsten et al., 2013) to 87.0 μmol O_2_ h^-1^ mg^-1^ chl a in *K. dissectum*.

The Klebsormidiophyceae genus *Interfilum* showed different kinetics of decrease in effective quantum yield under desiccation conditions in relation to their morphological characteristics (Karsten et al., 2014). While single celled strains exhibited a faster desiccation rate, cell packet forming strains showed slower desiccation rates (Karsten et al., 2014). In general, all investigated *Interfilum* strains exhibited optimum photosynthesis under low photon fluence rates, with no signs of photoinhibition under high light conditions up to 1500 μmol photons m^2^ s^-1^ (**Figure 5B**), (Karsten et al., 2014). In the additionally investigated Klebsormidiophyceaen genera *Entransia* and *Hormidiella* similar reactions to desiccation were observed (**Figure 5C**), (Herburger et al., 2016a). All these data suggest that Klebsormidiophyceae have low light requirements, coupled with no photoinhibition under higher irradiation, corroborating a high photophysiological plasticity.

In contrast, in Zygnematophyceae rETR curves show much higher initial saturation irradiance (*I*_k_ values). In the desmidiaceae *Cosmarium* (Stamenković and Hanelt, 2011), in the range of 1200 μmol photons m^2^ s^-1^ for tropical and cosmopolitan strains, somewhat lower in polar taxons (around 700 μmol photons m^2^ s^-1^). In a polar strain of *Zygnema I*_k_ values around 450 μmol photons m^2^ s^-1^ were observed (Holzinger et al., 2009). *I*_k_ values of *Zygnema* sp. collected in the Austrian Alps (~150 μmol photons m^2^ s^-1^) and in a lowland strain of *Zygnema* at ~450 μmol photons m^2^ s^-1^ (Herburger et al., 2015). The rETR curves showed significant difference in relation to the culture age, where the oldest cultures had a significant drop in the maximum electron transport rate (rETR_max_; **Figure 5D**), (Herburger et al., 2015). Signs of photoinhibition were observed in arctic and Antarctic *Zygnema* sp. strains, and the rETR_max_ values were drastically reduced upon osmotic stress (**Figures 5E,F**), (Kaplan et al., 2013). However, in general, the values determined for Zygnematophyceae point toward a higher light adaptation, and moreover remarkable resistance against UV irradiation has been observed (summarized in Holzinger and Lütz, 2006). How can this adaptation be realized?

The xanthophyll cycle pool size may contribute substantially to a well-developed photo protection process, in this way dissipating excessive irradiation (Stamenković et al., 2014). There is evidence for the increase in zeaxanthin content of the PS II antenna in high light tolerating *Cosmarium* strains (Stamenković et al., 2014). In *Zygnema* strains from arctic and Antarctic habitatas, a considerable amount of antheraxanthin and zeaxanthin was found (Pichrtová et al., 2013). The highest de-epoxidation state was found in an arctic strain, which went along with the highest concentration of phenolic compounds (Pichrtová et al., 2013).

These studies match well with the findings of high resistance to photoinhibition in an Antarctic strain of *Zygnema* sp. (Thangaraj, 2016). In this study the cells have been exposed to 400, 1400, 2100, and 3500 μmol photons m^-2^ s^-1^. After an initial drop of the *F*_v_/*F*_m_ value, the cells showed a rapid tendency to recover, even after unrealistically high irradiation. The authors claim some constitutive and genetically fixed preservation mechanisms responsible for the high tolerance in the Antarctic *Zygnema* sp. strain. Interestingly, d*F*_o_ did not recover to pre-treatment values, which indicated rearrangements of the LHC II complex.

Zygnematophyceae have shown remarkable stress tolerance to experimental UV radiation (Meindl and Lütz, 1996; Lütz et al., 1997). UV-effects in algae were summarized in Holzinger and Lütz (2006). In a study with field collected samples of *Zygnema* sp. from Svalbard, virtually no effect on the ultrastructure but a significant decrease of the *F*_v_/*F*_m_ value after 24 h UV A, but not after UV AB exposures was found (Holzinger et al., 2009). Similar results were gained by Pichrtová et al. (2013), albeit significant reduction in *F*_v_/*F*_m_ were observed in an arctic and an Antarctic strain of *Zygnema* sp. after a predominant UV A treatment for 8 days. Germ et al. (2009) did not find a decrease in *F*_v_/*F*_m_ after UV B exposure in an arctic *Zygnema* strain. In *Cosmarium* UV A radiation caused a twofold larger depression of *F*_v_/*F*_m_ when compared to that under PAR irradiation (Stamenković and Hanelt, 2014). The observation that in several of the investigated Zygnematophyceae UV A lead to more pronounced photoinhibition, might be ecologically relevant, as in natural sunlight the proportion of UVA is at least ten times higher than UV B, and moreover this waveband is not blocked by the ozone layer.

## Production of Protective Substances

Algae produce a vast spectrum of metabolites that help them to protect the cells against environmental stress. Some are produced as a result of stress but some are also produced constitutively. Precise classification according to their function is difficult, because very often one substance is involved in various processes. Here, we focus on two major protective mechanisms provided by accumulated compounds – osmotic acclimation, which is important for tolerating limiting water availability and UV (PAR) screening that protects exposed cells.

### Accumulation of Organic Osmolytes

Preventing water loss and maintaining homeostasis under stress conditions is essential for normal cellular function. Increasing salinity, desiccation, and freezing lead to osmotic dehydration and a reduced cellular water potential (Bisson and Kirst, 1995). Therefore, the physiological effects of these stresses are similar and the acclimation during exposure to an individual stress can result in resistance to other stresses (Morison and Sheath, 1985; Welsh, 2000; Vilumbrales et al., 2013). Nevertheless, these stresses are complex and components other than osmotic dehydration play a role in their effects (e.g., ionic component of the salt stress, Darehshouri and Lütz-Meindl, 2010). Recently osmotic stress induced ultrastructural changes leading to degradation of dictyosomes were investigated by TEM and FIB-SEM tomography in the charophyte green algae *Micrasterias* and *Nitella* (Lütz-Meindl et al., 2015). During the osmotic acclimation in response to long term hyperosmotic conditions, algae synthesize and accumulate various substances that increase the cellular osmotic value, leading to a negative osmotic potential (Bisson and Kirst, 1995). Organic osmolytes also play other roles in cellular protection. They can act as compatible solutes that are highly soluble, accumulate to high concentrations without interacting with cellular functions, protect proteins and stabilize membranes (Bisson and Kirst, 1995; Yancey, 2005). They can also act as antioxidants, cryoprotectants and heat protectants and can be rapidly degraded and used as respiratory substrates (Welsh, 2000; Yancey, 2005). Organic osmolytes are chemically diverse, comprising sugars, polyols, amino acids, and their derivatives (Welsh, 2000; Yancey, 2005).

Several studies reported osmolyte accumulation in various species of *Klebsormidium*. Sucrose and glutamic acid were detected as major osmoregulatory compounds of *K. flaccidum* and *K. sterile* subjected to osmotic stress in artificial seawater (Brown and Hellebust, 1980). *K. flaccidum* was capable to increase freezing tolerance during cold acclimation in a similar way to higher land plants (Nagao et al., 2008). The authors showed accumulation of sugars (mainly sucrose and glucose), but also of other compounds, such as amino acids and an unknown glycoside (Nagao et al., 2008). Kaplan et al. (2012) detected various soluble carbohydrates in two species of *Klebsormidium* (**Table 1**). The major proportion of oligosaccharides remained unidentified, the rest was dominated by raffinose, sucrose, glucose, xylose, galactose, mannose, inositol, fructose, glycerol, mannitol, and sorbitol (Kaplan et al., 2012). However, the total content of soluble carbohydrates was only approximately 1.2% of the dry weight (Kaplan et al., 2012). Karsten and Rindi (2010) reported sucrose as the major organic osmolyte of *Klebsormidium* sp. isolated from an urban wall in Germany. Sucrose accumulated in the cells with increasing salinity, in the highest salinity levels tested, however, the sucrose content and maximum quantum yield of PSII rapidly declined. This rather stenohaline response can be explained by the inability of Streptophyta to accumulate polyols as organic osmolytes (in contrast to most aeroterrestrial Chlorophyta, e.g., Gustavs et al., 2010), because sucrose cannot be accumulated to such a high content (Karsten and Rindi, 2010; Karsten et al., 2010). The only known report of sorbitol accumulation to high cellular concentrations by *K. marinum* (Brown and Hellebust, 1980) has to be viewed critically as molecular phylogenetic analyses revealed its position under Chlorophyta which resulted in its reclassification as *Stichococcus deasonii* (Neustupa et al., 2007).

**Table 1:** Summary of protective substances avoiding abiotic stress in charophyte green algae.

Charophyte Genus	Organic osmolytes	MAA	Phenolic compounds	Reference
*Klebsormidium*, *Hormidiella*		MAA 324 nm		Kitzing et al., 2014; Kitzing and Karsten, 2015
*Klebsormidium*	Sucrose, glucose, raffinose, xylose, galactose			Nagao et al., 2008; Karsten and Rindi, 2010; Kaplan et al., 2012
*Zygnema*	Sucrose, traces of glucose, fructose, mannitol			Hawes, 1990
*Ancylonema*			Purple vacuolar pigment	Remias et al., 2012a
*Mesotaenium*			Purpurogallin derivatives	Remias et al., 2009, 2012b
*Zygogonium*			Glycosylated gallic acid derivatives, complexed with iron	Aigner et al., 2013; Newsome et al., 2013; Herburger et al., 2016b
*Spirogyra*			Gallotannins	Nishizawa et al., 1985
*Zygnemopsis*			Unspecified	Figueroa et al., 2009
*Zygnema*			Unspecified	Pichrtová et al., 2013

Recent transcriptomic study of *K. crenulatum* showed up-regulation of sucrose synthase and sucrose phosphate synthase after desiccation stress (Holzinger et al., 2014) even though this enzyme has a different structure than in plants (Nagao and Uemura, 2012). In addition, several enzymes involved in the biosynthesis of the raffinose family of oligosaccharides were also up-regulated (Holzinger et al., 2014).

Low cellular osmotic potentials (-0.8 to -1.67 MPa) and tolerance to osmotic stress was also reported in arctic and Antarctic *Zygnema* spp. (Kaplan et al., 2013; Pichrtová et al., 2014a). Soluble sugars accounted for 9.9 ± 0.8% ash-free dry weight of field collected Antarctic *Zygnema* and 95% of the extracted sugars were identified as sucrose, with traces of glucose, fructose, and mannitol (Hawes, 1990). However, the author argued that this concentration is still too low to significantly depress the freezing point of the cell contents (Hawes, 1990).

Even though many streptophytes were shown to tolerate osmotic, desiccation and freezing stress, production of organic osmolytes within this group remains largely unknown or it seems that the detected organic osmolytes do not accumulate to high concentrations enough to fully explain the alga’s stress tolerance (Hawes, 1990; Kaplan et al., 2012). Therefore, it seems that accumulation of soluble carbohydrates alone cannot explain the high osmotic values and water stress tolerance of aeroterrestrial streptophytic algae.

### UV (and PAR) Screening Compounds

Strictly freshwater Charophyceae were found to be sensitive to UV B radiation, but at the same time they do not produce any UV absorbing compounds. They have to rely on natural UV attenuation provided by water and substances dissolved within (de Bakker et al., 2005).

Mycosporine-like amino acids (MAAs) are the most widespread compounds with UV screening function, commonly accumulating in many groups of algae and other organisms. They are colorless, water-soluble substances with peak absorbances between 310 and 360 nm (Cockell and Knowland, 1999; Shick and Dunlap, 2002). They can function also as antioxidants (Coba et al., 2009; Carreto and Carignan, 2011) and are involved in osmotic regulation (Shick and Dunlap, 2002; Oren, 2007). Within Charophyta, their production has been so far proven only in Klebsormidiophyceae. *K. fluitans* was shown to synthesize a so far unidentified MAA with absorption maximum at 324 nm (**Table 1**). After UV exposure, this compound was accumulated up to more than 1% DW (Kitzing et al., 2014). A closely related genus *Hormidiella* that occurs in similar habitats as *Klebsormidium* contains the same compound, while this compound was not present in *Entransia*, an aquatic genus from the same class. *Entransia* occurs in peat bogs and humic lakes where UV screening is provided by dissolved organic carbon of the water (Kitzing and Karsten, 2015).

Another group of substances with UV screening function are phenolic substances. They are produced by only few algal classes and within charophytes their production has been so far proven only in Zygnematophyceae (**Table 1**), which chemotaxonomically support their close relationship to land plants. Like MAAs, phenolics are water soluble and natural UV screens because of their aromatic groups. In addition, some phenolic substances cause strong pigmentation of the vacuoles and so they protect photosynthetic apparatus also from excessive PAR irradiation in a similar way as secondary carotenoids of some chlorophyte algae. In contrast to MAAs they do not contain nitrogen which makes their synthesis “cheaper” and therefore advantageous in extreme habitats with nutrient deficiency (Carreto and Carignan, 2011). This is probably the main reason why Zygnematophyceae are able to thrive in extreme environments like glacier surface where strong irradiation is coupled with very oligotrophic conditions. Phenolics also act as antioxidants and may play a role also against herbivores or parasites. Antibacterial activity of methanol extract of *Spirogyra varians* is also attributed to phenolics (Cannell et al., 1988).

*Ancylonema nordenskioeldii* and *M. berggrenii*, common zygnematophytes of both Alpine and Arctic glaciers, have dark purple brownish peripheral vacuoles containing phenolic pigments (**Figures 2H,I**). Phenolic compounds of *M. berggrenii* were determined as purpurogallin derivates (Remias et al., 2009, 2012b). The pigments cause even macroscopically visible coloration that significantly decreases the albedo of the ice and thus potentially promotes the melting of the glacier, with consequences for the whole ecosystem and global climate (Stibal et al., 2012).

Purple morph of *Z. ericetorum* similarly accumulates several unusual phenolic substances in vacuoles (**Figures 2B–F**), (Aigner et al., 2013). These components are mainly gallic acid derivatives, and their structure has recently been investigated (Newsome et al., 2013). In this way, *Zygogonium* has gained a high tolerance to iron, as well as aluminum (Herburger et al., 2016b). Various gallotannins were also detected in a *Spirogyra* sp. (Nishizawa et al., 1985). *Spirogyra pratensis* was able to produce purple pigmentation in the presence of iron (Allen and Alston, 1959). A whole range of unspecified, phenolics with UV screening ability but lacking typical dark color were detected also in other zygnematophytes, such as *Zygnema* (Pichrtová et al., 2013) and *Zygnemopsis* (Figueroa et al., 2009).

## Molecular Investigations of Stress Related Processes

At the moment, only a few publications are available using ‘omics’ techniques to address the molecular pathways behind stress tolerance and adaptation in charophytes. Comparisons of plant molecular processes between the charophyte green algae *Coleochaete orbicularis* and *Spirogyra pratensis* were performed and revealed a closer similarity to *Arabidopsis thaliana* than to the chlorophyte *Chlamydomonas reinhardtii* (Timme and Delwiche, 2010). The authors used a list of genes identified by Graham et al. (2000) to be important for colonization of land and found solid hits and even true orthologs in *Coleochaete* and *Spirogyra* including, e.g., cellulosic cell walls and cytokinetic phragmoplasts. According to the body plan no hit for asymmetric cell division was found in *Spirogyra*, but two hits were obtained in *Coleochaete* (Timme and Delwiche, 2010). A transcriptomic study of severe desiccation stress in *K. crenulatum* demonstrated that most of the highest upregulated transcripts do not show any similarity to known proteins (Holzinger et al., 2014). Major physiological shifts upon desiccation were the up-regulation of transcripts for photosynthesis, energy production and ROS metabolism (Holzinger et al., 2014). Classical desiccation responses, like late embryogenesis abundant proteins (LEA) or proteins involved in early response to desiccation (ERD) as well as osmolyte production (raffinose family oligosaccharides RFO) were upregulated. In contrast, transcripts of cell division, DNA replication, cofactor biosynthesis, and amino acid biosynthesis were strictly down-regulated as a consequence of desiccation stress (Holzinger et al., 2014).

Recently the effect of ionizing radiation was investigated in an arctic strain of *Zygnema* sp. (Choi et al., 2015). The photosynthetic efficiency markedly decreased with a 5 kGy dose, and the cells showed serious damage. However, an increase in DPPH radical scavenging activity was found in gamma-irradiated cells. The study is insofar interesting, as changes in the protein expression levels were investigated by a proteomics approach (Choi et al., 2015). Photosynthesis related proteins were up- or down-regulated, confirming the impact of gamma irradiation on the photosynthetic process. Proteins related to DNA repair, quinone oxidoreductase, regulation of microtubules and cell wall biogenesis were found to be upregulated. This means, that arctic *Zygnema* cells showed the capacity to actively repair damages to DNA, which is likely an effect of ionizing radiation. The ubiquinone oxidoreductase 13-kD-like subunit was also upregulated upon gamma irradiation. This protein is a coenzyme of the mitochondrial NADP-dehydrogenase, essential for NAPH oxidation. Interestingly, the regulation of cytoskeletal elements, in the particular case through an armadillo repeat-containing kinesin protein, that is involved in the ATP-binding mechanism (Choi et al., 2015). This might interact with organelle positioning and cell wall deposition.

In contrast, energy metabolism, isoprene biosynthesis and protein biosynthesis related proteins were down-regulated with the applied gamma-irradiation (Choi et al., 2015). This can be considered as a protection mechanism.

## Algae and Microbe Interaction

There is increasing knowledge that charophytes serve as hosts for microbial communities that occur within surface mucilage matrices (Knack et al., 2015). These microbial associations were suggested to nurture the earliest plants and influence their biogeochemical roles. Microbiomes are known to be closely related to host organisms from certain lineages, it is hypothesized that microbiomes of early-diverging modern bryophytes and closely related charophyte green algae have commonalities reflecting the ancestral traits (Knack et al., 2015). Metagenomic sequence data were used from the late-diverging charophytes *Chaetosphaeridium* and *Coleochaete*, as well as the liverwort *Conocephalum* to infer bacteria and fungi for comparisons with the microbiome of an outgroup chlorophytic green alga *Cladophora*. It was found that the streptophyte (charophyte green algae and liverwort) microbiomes contained N-fixing cyanobacteria (*NifH* genes indicating nitrogen fixation), methanotrophs (*pMMo* genes indicating methane oxidation) and early diverging fungi. They were much more similar to each other than to the *Cladophora* microbiota (Knack et al., 2015).

Symbiotic interactions were also tested in a large scale transcriptomic analysis comparing 259 transcriptomes (10 green algal and basal land plant genomes) and were suggested to be involved in colonizing land by plants (Delaux et al., 2015). The appearance of a key regulator, a calcium and calmodulin-dependent protein kinase, suggested as symbiotic signaling pathway predated the conquering of land (Delaux et al., 2015). In their study, they tested the newly generated deep transcriptomes of the charophyte complex *Closterium peracerosum*-*strigosum*-*littorale* and the draft genome of *Spirogyra* sp. as well as transcriptomes of liverworts, mosses and hornworts. They report, that potential homologs of DMI1 and CCaMK (‘symbiotic genes’ belonging to the signaling module) were found in charophytes and even in chlorophytes (Delaux et al., 2015). This is insofar not surprising, as virtually all lichen forming algae (besides cyanophyta) belong to the chlorophytic lineage. Despite being very successful on land by tolerating extreme desiccation (e.g., Kranner et al., 2008), lichens did not give rise to further phylogenetic developments on land like the charophytes. The mutualistic fungal partners (Mucoromycotina/Glomeromycota), acting in some sort of primitive mycorrhizal symbiosis in early land plants, have recently been reviewed by Field et al. (2015).

## Function of Plant Hormones

**FIGURE 6:**
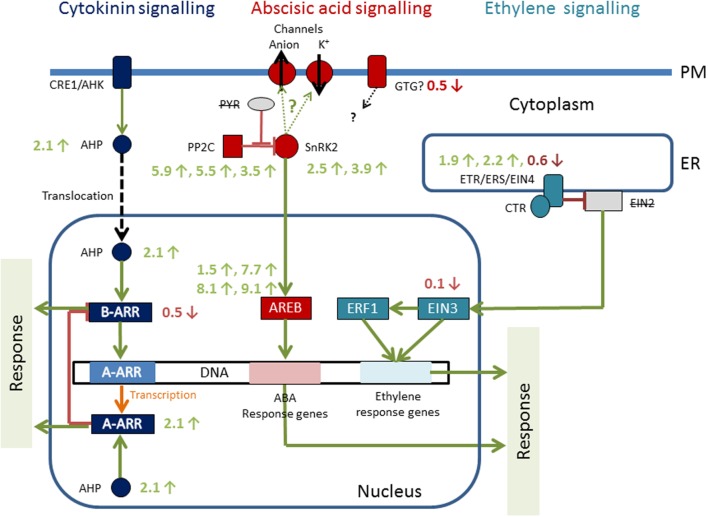
**Hormone signaling pathways of *Klebsormidium crenulatum*; numbers are transcription changes (-fold) up or down regulation upon severe desiccation stress.** Reprinted from Holzinger and Becker (2015).

Phytohormones play a critical role in signal transduction upon stress reactions. Recently transcriptomic and genomic datasets became available in the early branching *Klebsormidium* (Holzinger et al., 2014; Hori et al., 2014), which allow to analyze the role of phytohormones in stress sensing (**Figure 6**). With the KEGG pathway reconstruction tool, almost complete pathways were found for cytokinin signaling, abscisic acid (ABA) signaling and ethylene response in *K. crenulatum* (Holzinger and Becker, 2015). In the draft sequence of *K. flaccidum* genome, signaling pathways for auxin, ABA, cytokinin, salicylic acid, and JA were found (Hori et al., 2014). Particularly the ABA response is well known in abiotic stress reactions. When *K. crenulatum* cells are exposed to severe desiccation stress ABA signaling components PP2C and SnRK2 were significantly increased, together with the nuclear AREB protein (Holzinger and Becker, 2015). There is a 70% overlap in differential gene expression profiles between plants that have been stressed with desiccation and high light, and it has to be stated that the majority of the ABA is synthesized in the plastids itself (de Vries et al., 2016). However, experimental ABA application did not change cold tolerance in *K. flaccidum* (Nagao et al., 2008). Also, orthologs for the cytokinin receptors CRE1/AHK (in the plasma membrane), cytoplasmic AHP and the transcription factor A-ARP were upregulated as a consequence of desiccation stress in *K. crenulatum* (Holzinger and Becker, 2015). The abiotic stress of severe desiccation regulates the expression of three classical phytohormone pathways in *K. crenulatum*, corroborating that these early branching charophyte algae have the prerequisites for living on land (Holzinger and Becker, 2015). For successful terrestrialization, mechanisms to sense the external environment are crucial.

First evidence for ethylene biosynthesis and pathway genes in charophyte green algae have been given by Timme and Delwiche (2010). Ethylene response appears to be highly conserved as a plant hormone for the past 450 mio years (Ju et al., 2015). It was shown, that *Spirogyra* ethylene-signaling homologs have the capacity to partially rescue *Arabidopsis* defective mutants, indicating homologous ethylene-signaling pathways, implying that the common aquatic ancestor possessed this pathway already prior to the colonization of land (Ju et al., 2015).

## Strategies in an Evolutionary Point of View

In a recent review, Delwiche and Cooper (2015) give an outstanding overview on the evolutionary background of the origin of the terrestrial flora. The discrepancy between single-gene and morphological analysis and genome-scale phylogenetic analysis are discussed, the latter clearly indicating the Zygnematophyceae as sister taxon to land plants (Timme and Delwiche, 2010; Wodniok et al., 2011; Timme et al., 2012; Wickett et al., 2014; Zhong et al., 2014, 2015). Recently this view has additionally been supported by analysis of plastid evolution (de Vries et al., 2016). While many prerequisites for the terrestrial life-style are common in several groups of charophytes, only zygnematophyceaen algae share with land plants the transfer of a few plastid genes to the nucleus (de Vries et al. (2016). Chloroplasts became more submissive to nuclear control by key genes involved in the organelle division machinery (de Vries et al., 2016). Only basal charophyta (and some chlorophyta) possess the plastid division genes *ftsI*, *ftsW*, and *minD*. In contrast, ZCC-clade algae transferred these genes to the nucleus or lost them (de Vries et al., 2016). Interestingly, only the genus *Zygnema* possess *cysA* and *cysT* (encoding components in the sulfate ABC transporter system). These genes were not observed in any other zygnematophyceaen plastid genomes. Thus, the *Zygnema* plastid genome coding capacity was considered most similar to the hypothetical land plant ancestor (de Vries et al., 2016).

The link to understand evolution of terrestrial organisms from aquatic ones is to remember that life remains a fundamentally aquatic process (Delwiche and Cooper, 2015). The ancestral habitat for all charophyte lineages is freshwater, so terrestrial and semi-terrestrial environments are intermittently available to freshwater organisms. With the unpredictable changes of water availability organisms conquering land require desiccation tolerance mechanisms. Harholt et al. (2016) go even a step further, hypothesizing that charophyceaen green algal ancestors were already living on land for some time before the emergence of land plants. Their hypothesis is based on the idea that ancestral charophytes evolved their cell walls in response to the terrestrial selection pressure. However, this has to be seen critically, as strictly aquatic algae including chlorophytes have also evolved a complex cell wall modification machinery (e.g., Domozych et al., 2012). The occurrence of complex cell walls can thus not only be seen in connection with terrestrialization. Moreover, terrestrialization is not unique for charophytes evolutionary close to land plants, but also phylogenetic basal lineages contain terrestrial forms (e.g., chlorophytes like *Stichococcus*, *Trentepohlia*, *Apatococcus*, *Prasiola*).

Ecological differentiation has been suggested to trigger the formation of cryptic species in Klebsormidiales (Škaloud and Rindi, 2013). In this study, 14 lineages could be resolved based on concatenated ITS rDNA + *rbc*L data sets with clear preferences for either natural subaerial substrata, artificial subaerial substrata or aquatic habitats. These results were interpreted as an evidence for the existence of a high number of cryptic species within morphospecies (Škaloud and Rindi, 2013). These investigations were expanded to a global scale study in the same genus, where in addition to the global ubiquity strong biogeographic patterns were revealed on a local scale (Ryšánek et al., 2015). Again, for the local fine-scale structuring of the genotypes habitat differentiation was suggested. In a recent study by Ryšánek et al. (2016) one of these ecological factors – the pH value (pH 4 to pH 8) of the substrate was investigated, and hypothesized as a potential important factor for sympatric speciation.

Survival in vegetative state, e.g., in form of ‘pre-akinetes’ in *Zygnema* (Holzinger et al., 2009; Pichrtová et al., 2014a; Herburger et al., 2015) is important prerequisite for terrestrial life style. But also morphological features support this survival; One example is the holdfast of *Spirogyra* cells, which is preceded by callose deposition at the tip of developing rhizoids (Yamada et al., 2003). Branching is common in *Z. ericetorum* (Stancheva et al., 2014), and more complex morphologies can be observed in *Coleochaete*, still having the advantage of a small thallus (Leliaert et al., 2012). Structural simplicity might be advantageous in shallow and transient aquatic habitats, because even a thin film of water can provide enough room for coverage (Delwiche and Cooper, 2015). In addition, many species produce large amounts of mucilage with water holding capacities. The ultimate adaptation strategy to a terrestrial lifestyle is the sexual reproduction of Zygnematophyceae, which completely lacks flagellate stages and in the conjugation tube only gliding motility is necessary (Ikegaya et al., 2012).

In the present review, knowledge about stress tolerance mechanisms in Charophyta connected with life in terrestrial environment was summarized. We aimed to include insights derived from recent studies using omics techniques to investigate charophyte stress responses. A combination of methods will in future help to elucidate the stress tolerance that allows survival of this algal group under unfavorable conditions, and which key elements were responsible for the final transition to land.

## Author Contributions

All authors listed, have substantial, direct and intellectual contribution to the work, and approved it for publication.

## Conflict of Interest Statement

The authors declare that the research was conducted in the absence of any commercial or financial relationships that could be construed as a potential conflict of interest.
